# Patientenbezogene Ergebnisindikatoren (PROMs) bei erwachsenen Cochleaimplantatpatienten

**DOI:** 10.1007/s00106-024-01510-2

**Published:** 2024-09-11

**Authors:** Stefan Weder, Marco D. Caversaccio, Georgios Mantokoudis

**Affiliations:** Universitätsklinik für Hals‑, Nasen- und Ohrenkrankheiten, Kopf- und Halschirurgie, Inselspital, Freiburgstr. 20, 3012 Bern, Schweiz

**Keywords:** Cochlea-Implantat, Patientenbezogene Ergebnisindikatoren (PROMs), Lebensqualität, Hörverlust, Sprachverständlichkeit, Cochlear implant, Patient-related outcome indicators, Quality of life, Hearing loss, Speech intelligibility

## Abstract

**Hintergrund:**

Cochleaimplantate (CI) bieten Personen mit schwerer sensorineuraler Hörschädigung die Möglichkeit einer artifiziellen Hörwahrnehmung. Die standardisierte Erhebung von Sprachverständlichkeitstests ist weit verbreitet, während die systematische Erfassung von patientenbezogenen Ergebnisindikatoren (PROMs) noch uneinheitlich ist.

**Methodik:**

Für die Thematik relevante PROMs-Instrumente wurden basierend auf den Kriterien Verbreitung, Klarheit und Relevanz evaluiert und ausgewählt, in den klinischen Alltag integriert und an longitudinalen Zeitpunkten getestet.

**Ergebnisse:**

Drei PROMs-Instrumente wurden ausgewählt und erfolgreich in die klinische Routine integriert. Der Vergleich von 2 Messzeitpunkten von 25 Individuen zeigten Verbesserungen im subjektiven Sprachverstehen und der Tinnitus-Wahrnehmung.

**Schlussfolgerung:**

Unsere Studie zeigt die klinische Umsetzung und Integration von PROMs bei erwachsenen CI-Kandidaten und -Patienten. PROMs sind ein vielversprechendes Instrument zur Unterstützung in verschiedenen Behandlungsphasen, sowohl als Entscheidungshilfe für potenzielle CI-Kandidaten als auch zur Überwachung nach der Implantation.

## Abstract (en)

**Background:**

Cochlear implants (CI) provide individuals with severe sensorineural hearing loss the opportunity for artificial auditory perception. The standardized documentation of speech intelligibility tests is widespread, while the systematic capture of patient-related outcome measures (PROMs) remains inconsistent.

**Methodology:**

Relevant PROM instruments were evaluated and selected based on the criteria of dissemination, clarity, and relevance, integrated into routine clinical practice, and tested at longitudinal time points.

**Results:**

A total of three PROM instruments were selected and successfully integrated into the clinical routine. The comparison of 2 measurement points from 25 individuals showed improvements in subjective speech comprehension and tinnitus perception.

**Conclusion:**

This study demonstrates the clinical implementation and integration of PROMs in adult CI candidates and patients. The PROMs are a promising tool to support various phases of treatment, both as a decision aid for potential CI candidates and for monitoring after implantation.

Cochleaimplantate (CI) ermöglichen Personen mit schwerer sensorineuraler Hörschädigung eine artifizielle auditorische Wahrnehmung [6]. Bei der Indikationsstellung verfolgen wir 2 Hauptziele: Einerseits soll das Implantat die Wahrnehmung der Lautsprache wieder ermöglichen, andererseits soll die Lebensqualität von Betroffenen gesteigert werden. Während für das Sprachverstehen klar definierte und validierte Testbatterien etabliert sind, variiert die systematische Erhebung von patientenbezogenen Ergebnisindikatoren je nach Implantationszentrum – teilweise findet sich gar nicht statt.

## Hintergrund

Bei den Testbatterien für das Sprachverstehen haben sich im deutschsprachigen Raum folgende Instrumente durchgesetzt: der Freiburger Sprachverständlichkeitstest [12] und der Oldenburger Satztest [19]. Die Probanden müssen dabei akustisch präsentierte Worte und Sätze wiedergeben. Es wird entweder der Prozentsatz korrekt verstandener Worte berechnet oder der Schallpegel ermittelt, bei dem 50 % der Worte korrekt wiedergegeben werden können. Die Testungen sind sowohl mit als auch ohne Hörgeräteversorgung sowie vor und nach einer Implantation durchführbar. Normwerte ermöglichen die Einschätzung der Befunde und die Vergleichbarkeit des Therapieerfolgs zwischen verschiedenen Individuen, Krankheitsbildern und Implantationszentren.

Anders verhält es sich bei den patientenbezogene Ergebnisindikatoren, im englischsprachigen Raum als PROMs („patient-related outcome measures“) bekannt. Diese Befragungsinstrumente erfassen subjektive Informationen über den Gesundheitszustand, die Lebensqualität und die individuellen Erfahrungen vor und nach einer Behandlung. Sie ermöglichen es, den Einfluss medizinischer Interventionen aus Patientenperspektive zu beurteilen. Im deutschsprachigen Raum besteht noch keine Einigkeit darüber, welche Fragebögen sich für CI-Kandidaten und -Träger am besten eignen. Es existieren verschiedene Instrumente zur Bewertung von Sprachverstehen, Höranstrengung, Klangwahrnehmung, Ohrgeräuschen und Schalllokalisierung [6, 9, 17, 18, 21].

Zusammenfassend lässt sich festhalten, dass Sprachverständlichkeitstests den Therapieerfolg nach einer Implantation nicht vollständig abbilden. Aus diesem Grund ist die standardisierte Erhebung von patientenbezogenen Ergebnisindikatoren unerlässlich. Dies ermöglicht eine umfassendere und individuellere Bewertung des Ergebnisses vor und nach einer Implantation. Zudem kann durch die Erhebung vor der Indikationsstellung die Beratung von Betroffenen optimiert werden, indem ihr Leidensdruck besser verstanden wird.

Das Ziel der hier vorgestellten Arbeit war es, zu ermitteln, welche PROMs sich für eine effiziente Integration bei der Abklärung und Nachsorge von CI-Kandidaten und -Patienten eignen. Anschließend sollen die Instrumente im klinischen Alltag getestet werden.

## Methodik

**Abb. 1:**
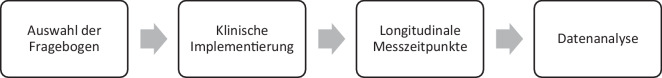
Studienablauf

Unsere Studie setzte sich aus 4 Schritten zusammen (Abb. 1).

Zuerst wählten wir PROMs-Instrumente aus, indem wir Kriterien wie Verbreitung, Validierung, Kürze (maximal 15 Fragen) und Klarheit berücksichtigten. Empfehlungen des ICHOM (International Consortium of Outcome Measurements) wurden, wenn verfügbar, ebenso einbezogen. Die Auswahl der PROMs beschränkte sich auf erwachsene, deutsch- oder französischsprachige Personen. Die Instrumente mussten folglich in validierter deutscher und französischer Form vorliegen und von den Betroffenen als relevant betrachtet werden. Ein Betroffenenverband unterstützte uns dabei, Angemessenheit und Verständlichkeit zu überprüfen.

Nach der Auswahl wurden die Fragebögen in den klinischen Alltag eingebettet. Wir legten Wert darauf, die Befragung effizient und elektronisch durchzuführen, um den Verwaltungsaufwand zu minimieren und eine präzise Datenerhebung zu sichern.

Die ausgewählten Fragebogen wurden an Individuen abgegeben, die zur CI-Abklärung an unsere Spezialsprechstunde verwiesen wurden (präoperative Befragung). Postoperativ wurden die Erhebungen an routinemäßig geplanten Einstellungsterminen angepasst.

Für die Datenanalyse der setzten wir deskriptive Methoden und nichtparametrische Tests („Wilcoxon paired test“) ein, um die Veränderungen vor und nach der Operation zu vergleichen. Ein *p*-Wert unter 0,05 galt als signifikant. Neben den PROMs analysierten wir auch audiologische Daten. Bei den Hörtestungen wurden das Reintonaudiogramm für das betroffene Ohr analysiert. Dabei wurde jeweils der Mittelwert der Reintöne (PTA, „pure tone average“) für die Frequenzen 500 Hz (Hertz), 1 kHz und 2 kHz berechnet [27]. Die individuellen Hörstörungen wurden gemäß dem PTA des Gegenohrs in bilateral (PTA Gegenohr > 55 dB HL [„decibel hearing level“]), asymmetrisch (PTA Gegenohr > 30 and ≤ 55 dB HL) und unilateral (PTA Gegenohr PTA ≤ 30 dB HL) eingeteilt. Bei den Sprachaudiogrammen (Stimulationspegel 65 dB SPL [„decibel sound pressure level“]) wurde das präoperative Verstehen von Freiburger Ein- und Zweisilbern mit Hörgeräteversorgung sowie das entsprechende Sprachverstehen 6 Monate nach Implantation mit aktivem Sprachprozessor ermittelt. Wir führten eine lineare Regression durch, um den Zusammenhang zwischen dem SSQ(Speech, Spatial and Quality of Hearing Questionnaire)-12-Fragebogen und den Sprachverständlichkeitstests zu ermitteln.

## Resultate

### Evaluation und Applikation der PROMs-Instrumente

Bislang steht kein spezifisches Set des ICHOM in diesem Zusammenhang zur Verfügung [29]. Im Bereich der Hörstörungen, der Hörimplantate und deren assoziierten Problemen im Alltagsleben gibt es diverse Teilbereiche und entsprechend viele mögliche Befragungsinstrumente [7, 24, 25]. Wir entschieden uns bei der Implementierung für 3 Teilbereiche: i) allgemeine Gesundheit, ii) Sprach- und Hörwahrnehmung sowie iii) Tinnitus, mit jeweils einem Instrument. Nach Literaturrecherche und Absprache mit dem Betroffenenverband wurden folgende 3 PROMs-Werkzeuge ausgewählt:

Für die Bewertung der allgemeinen Gesundheit wählten wir den PROMIS-10-Fragebogen aus [15]. Dieser Fragebogen wird an unserer Institution fachübergreifend seit Jahren verwendet und erlaubt entsprechende Vergleichswerte. Zudem ist die Bearbeitungsdauer kurz (unter 5 min), die Fragen sind klar und einfach, und es besteht eine Validierung hinsichtlich körperlicher und psychischer Gesundheit in verschiedenen Patientenpopulationen [2, 4]. Für die Bewertung der Sprach- und Hörwahrnehmung wählten wir den SSQ-12 [26] aus. Der Test ist in Fachkreisen bekannt und wird weltweit verwendet. Zudem verglichen Grundman et al. bezüglich Sprachverstehen prospektiv unterschiedliche Instrumente und schlugen den SSQ-12 als bevorzugtes Instrument vor [11]. Im Bereich der Tinnitusbewertung wählten wir den TQ(Tinnitus Questionnaire)-12 aus [10, 16]. Dieses Instrument wird bereits seit Jahren von den 5 Schweizer CI-Zentren verwendet. Das Instrument zeigt eine ausgezeichnete Test-Retest-Reliabilität und eine Ausrichtung auf longitudinalen Daten, was ihn für die Bewertung therapeutischer Interventionen wie der Cochleaimplantation geeignet macht [10].

Wir definierten 3 Messpunkte für die PROMs:

- präoperativ zur Erfassung des Leidensdrucks und Unterstützung der Entscheidungsfindung,
- 6 Monate nach der Operation zur Bewertung der Anpassung an das elektrische Hören und Verbesserung des Sprachverstehens und
- 24 Monate postoperativ zur Beurteilung der Langzeitergebnisse und Stabilisierung des Hörerlebnisses.

Diese Zeitpunkte sind an die regulären Prozessoreinstellungen und Kontrollen gekoppelt.

**Abb. 2:**
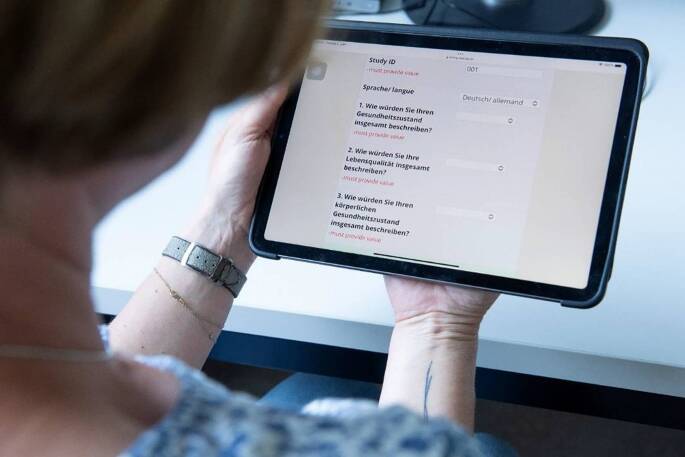
Die 3 Befragungsinstrumente werden elektronisch erfasst. Sie sind sowohl in deutscher als auch in französischer Sprache verfügbar

Wir konnten die ausgewählten Fragebogen in jeweils deutscher und französischer Fassung in elektronischer Form auf der Redcap Datenplattform aufsetzen [13]. Die Fragebogen wurden dabei auf einem Tablet präsentiert (Abb. 2). Die Befragung konnte nur abgeschlossen werden, wenn alle Fragen beantwortet wurden. Bei Unklarheiten hatten die Betroffenen die Möglichkeit, sich an das ärztliche Personal zu wenden.

### Studienpopulation

Die Datenerhebung begann im Januar 2022 und wird seitdem kontinuierlich fortgesetzt. Alle in den Resultaten aufgeführten Patienten erteilten schriftlich ihr Einverständnis zur Verwendung ihrer Daten für Forschungszwecke. Seit Messbeginn wurden die PROMs-Erhebung bei 91 Individuen begonnen (mindestens ein Messzeitpunkt). Bei 25 Individuen lagen 2 Messzeitpunkte vor. Ein Individuum erreichte den letzten Messzeitpunkt nach 24 Monaten. Da die Befragung in den klinischen Ablauf integriert wurde (Abgabe der Fragebogen vor den Sprechstundenterminen), lag die Rücklaufquote bei 100 %.

Für die nachfolgenden Analysen wurden nur Patienten eingeschlossen, die 2 Messzeitpunkte aufwiesen (präoperativ und 6 Monate postoperativ; insgesamt 25 Individuen, 13 Frauen und 12 Männer). Die demographischen Daten und die der Hörtestungen zeigt Tab. 1.

**Tab. 1:** Demographische Daten der Studienpopulation, welche den Zeitpunkt t2 erreicht hat

ID	Geschlecht	Alter bei OP	Seite	Sprache	Ätiologie	Hörverlust	PTA prä	PTA post	ES prä	ZS prä	ES post	ZS post
01	w	61	Rechts	D	Hörsturz	U	105	107	0	0	40	100
02	w	29	Links	F	Kongenital	B	93	117	0	0	70	95
03	m	54	Rechts	D	Otosklerose	A	120	120	0	0	45	100
04	m	57	Links	D	M. Menière	B	75	112	15	0	85	100
05	m	59	Links	F	M. Menière	B	68	108	10	50	40	70
06	m	42	Rechts	D	Kongenital	B	107	117	0	25	0	25
07	w	68	Links	D	Hörsturz	B	83	90	5	30	65	100
08	m	75	Rechts	D	Progressiv	B	73	110	0	70	70	100
09	m	75	Rechts	D	Trauma	A	120	112	0	0	45	90
10	m	90	Links	D	Progressiv	B	97	120	0	20	45	100
11	m	76	Links	D	Progressiv	B	85	95	0	0	85	100
12	w	69	Rechts	D	Trauma	A	70	120	0	0	30	100
13	w	40	Rechts	D	Hörsturz	U	77	105	0	0	35	100
14	m	28	Rechts	F	Trauma	U	115	120	0	0	85	100
15	w	78	Rechts	D	Ohrinfekte	B	117	120	0	0	45	95
16	w	33	Rechts	F	Progressiv	B	80	125	0	0	0	30
17	w	49	Rechts	F	Otosklerose	B	120	120	0	0	55	85
18	w	76	Links	D	Hörsturz	A	120	120	0	0	35	85
19	w	59	Rechts	D	Hörsturz	U	82	102	0	0	55	100
20	m	62	Links	D	M. Menière	A	82	120	0	0	40	100
21	w	54	Links	D	Meningitis	B	115	120	0	20	85	100
22	w	65	Rechts	D	Hörsturz	B	78	97	0	10	85	100
23	w	48	Rechts	D	Progressiv	B	117	120	0	0	85	100
24	m	78	Rechts	D	Progressiv	B	65	120	0	0	80	100
25	m	63	Rechts	D	Hörsturz	B	103	113	0	0	85	100

*ES* Freiburger Einsilber-Verstehen in Prozent bei 65 dB SPL („decibel sound pressure level“), *Hörverlust A* asymmetrischer Hörverlust (Gegenohr: PTA > 30 and ≤ 55 dB HL), *Hörverlust B* bilateraler Hörverlust (Gegenohr: PTA > 55 dB HL), *Hörverlust U* unilateraler Hörverlust (Gegenohr: PTA ≤ 30 dB HL), *OP* Operation, *Prä* präoperativ, *PTA* „pure tone average“, *post* postoperativ, *ZS* Freiburger Zweisilber-Verstehen in Prozent bei 65 dB SPL, *D* Deutsch, *F* Französisch

### Analyse der der PROMs-Instrumente

**Abb. 3:**
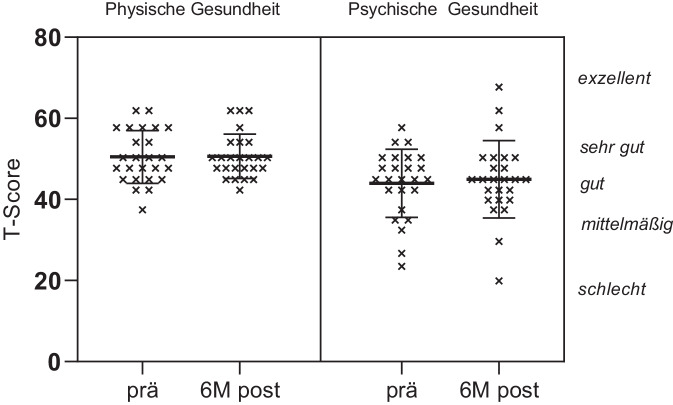
PROMIS-10-Ergebnisse: Werte für physische und psychische Gesundheit vor und 6 Monate nach Operation. T‑Werte auf *linker X‑Achse*, Beschreibungen auf *rechter X‑Achse*. *Dicker Balken* Mittelwert, *Whisker* Standardabweichung

Beim PROMIS-10-Fragebogen werden 2 zusammenfassende Werte berechnet: ein Wert für die körperliche Gesundheit und ein Wert für die psychische Gesundheit. In unserer Kohorte lag die psychische Gesundheit im Vergleich zur physischen Gesundheit leicht tiefer. Beide Werte waren aber vergleichbar mit denen der durchschnittlichen Normalbevölkerung [27]. Postoperativ gab es für beide Werte keine signifikante Veränderung. Interessant war die Einzelfrage „soziale Aktivitäten“. Dort verbesserte sich der Score 6 Monate nach Implantation von 3 auf 3,3 Punkte bei einem Maximum von 5 Punkten. Auch dieses Ergebnis war statistisch nicht signifikant (Abb. 3).

**Abb. 4:**
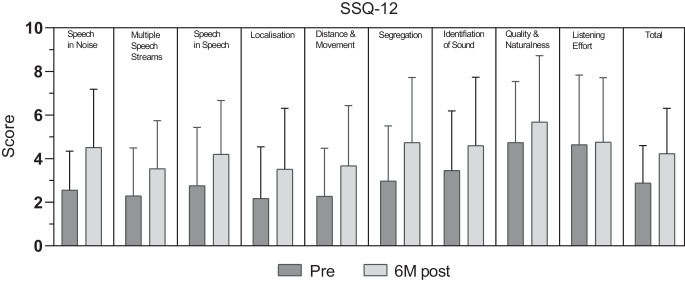
SSQ(Speech, Spatial and Quality of Hearing Questionnaire)-12-Ergebnisse: Skalenwerte auf einer visuellen Analogskala von 0–10 Punkten für die 9 Unterskalen und die Gesamtveränderung, vor und 6 Monate nach Implantation

Der SSQ-12 besteht aus 12 Fragen, die 9 Unterskalen bilden: Sprache in Ruhe, Sprache im Lärm, Sprache in Sprachkontexten, Hören mehrerer Sprachströme, Lokalisierung, Entfernung und Bewegung, Trennung, Identifizierung von Geräuschen, Qualität und Natürlichkeit sowie Höraufwand [26]. In unseren Resultaten verbesserten sich im Mittel 8 Subskalen. Am deutlichsten war die signifikante postoperative Zunahme bei der Frage „Sprachverstehen im Lärm“ (Abb. 4, *p* = 0,038, korrigiert). Eine Verbesserung zeigte sich auch bei Fragen zum Sprachverstehen in Räumen mit anderen sprechenden Personen (*p* = 0,039, unkorrigiert), wenn unterschiedliche Personen nacheinander sprechen (*p* = 0,018, unkorrigiert), oder zu Geräuschdiskrimination (*p* = 0,028, unkorrigiert), allerdings waren die statistischen Werte nach Korrektur für multiples Testen nicht mehr signifikant. 6 Monate nach Cochleaimplantation zeigte sich zudem noch keine signifikante Verbesserung bei Fragen zur Klang-Identifikation, Klang-Natürlichkeit und Höranstrengung. Mittels linearer Regression konnten wir auch keinen signifikanten Zusammenhang zwischen den einzelnen Elementen des SSQ-12 und dem postoperativen Sprachverstehen (Einsilber) ermitteln.

**Abb. 5:**
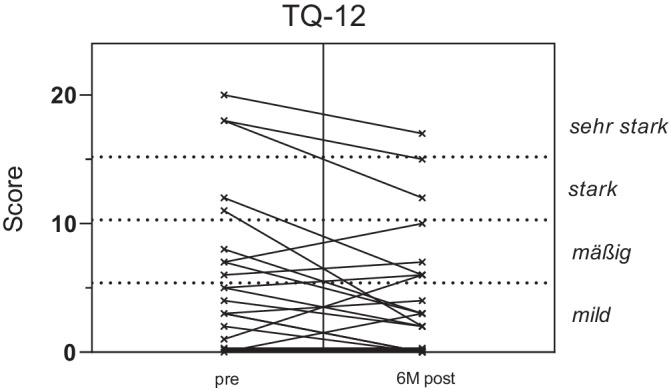
TQ-12-Ergebnisse: Tinnitus-Schweregrade vor und 6 Monate nach Operation. Score auf linker Y‑Achse, Beschreibungen auf rechter Y‑Achse

Für den TQ-12 können auf der Grundlage einer Quartilsberechnung vier Schweregrade berechnet werden: leicht, mittelschwer, schwer und sehr schwer [16]. Die meisten Studienteilnehmer wiesen präoperativ keinen (*n* = 6) oder einen milden Tinnitus (*n* = 11) auf. Wenige Individuen waren von einem mittelgradigen (*n* = 4), schwergradigen (*n* = 3) oder schwerstgradigen Tinnitus (*n* = 2) betroffen. Bei der Mehrzahl der Patienten verbesserte sich der Tinnitus 6 Monate nach Implantation (*n* = 14, mittlere Abnahme −4,8 Punkte). Bei 4 von 6 Patienten, die vor Implantation keine Beschwerden hatten, blieb dies auch nach Implantation so. Bei 11 Patienten nahmen die Tinnitusbeschwerden 6 Monate nach der Operation zu (im Mittel 2,3 Punkte). Die Befunde sind in der Abb. 5 dargestellt.

## Diskussion

Unsere Studie zeigte die erfolgreiche klinische Umsetzung und Integration von PROMs bei erwachsenen CI-Kandidaten und -Patienten. Um den Erfolg der Cochlea-Implantation zu quantifizieren, verwenden die meisten Zentren heutzutage normierte Sprachverständlichkeitstest in Ruhe oder Störlärm. Im Gegensatz dazu wird die Bewertung des subjektiven Nutzens nicht standardisiert erhoben. Es ist bekannt, dass der subjektive Nutzen einen direkten Einfluss auf die Geräteakzeptanz, die tägliche Tragedauer und die Motivation der Patienten hat. Hohe Erwartungen in Kombination mit einer geringen Patientenzufriedenheit und Motivation sind bekannte Faktoren für schlechtere Ergebnisse und höhere Raten der Nichtnutzung des Implantats, insbesondere bei Patienten mit einseitiger Taubheit [28]. Aus diesem Grund sollten patientenzentrierte Faktoren in der Abklärung wie auch in der Nachsorge eine zentrale Rolle erhalten.

Gemäß Aaronson et. al können PROMs für folgende Zwecke verwendet werden: als Screening-Tool, als Monitoring-Tool, zur Entscheidungshilfe möglicher Behandlungen, für die Team-Kommunikation und bei der Überprüfung der Behandlungsqualität [1]. PROMs könnten im Zusammenhang mit CIs in unterschiedlichen Phasen des Behandlungsprozesses Anwendung finden. So könnten diese als zusätzliche Entscheidungshilfe potenzielle CI-Kandidaten unterstützen oder nach erfolgter Implantation den subjektiven Therapieerfolg abbilden. Ferner könnten PROMs die Kommunikation innerhalb des Behandlungsteams vereinfachen, wodurch ein besser abgestimmtes und effektiveres Behandlungskonzept ermöglicht wird. Schließlich könnten diese die Qualität der Versorgung nachvollziehbar bewerten. Durch die longitudinalen Datenpunkte können der Hörverlust vor Implantation sowie die frühe und spätere Rehabilitationsphase abgebildet werden.

### Auswahl und Implementierung der PROMs

Die final gewählten PROMs-Instrumente wurden als klar und alltagsrelevant bewertet und erfordern weniger als 15 min zum Ausfüllen, was während regulärer klinischer Nachsorge elektronisch erfolgt, ohne zusätzliche Termine für Patienten. Seit ihrem Start im Januar 2022 hat sich an unserem Zentrum die Erhebung der PROMs fest in den klinischen Alltag integriert, erlaubt die kontinuierliche Generierung von Normwerten für CI-Kandidaten und Träger und verbessert somit die patientenspezifische Beratung und Behandlungsbeurteilung. Sie ergänzen die Sprachverständlichkeitstests und tragen zur qualitativen Bewertung des therapeutischen Erfolgs bei.

### Prä- und postoperative Hörtestungen

Das residuelle, akustische Gehör verschlechterte sich 6 Monate nach Implantation um 17 dB HL. Während der gleichen Zeitspanne verbesserte sich das Sprachverstehen bei den Zwei- und Einsilbern im Mittel um 57 % (*p* < 0,001) bzw. 83 % (*p* > 0,001). Diese Werte sind vergleichbar mit bereits publizierten Daten aus einer multizentrischen Studie [3]. Wir können somit festhalten, dass sich unsere Population nicht wesentlich von Vergleichspopulationen unterscheidet und sich somit eignet, patientenbezogene Ergebnisindikatoren zu untersuchen.

### PROMs

Der globale und psychische Gesundheitszustand blieb in unserer Studienpopulation 6 Monate nach der Operation unverändert. Mögliche Erklärungen hierfür sind die kleine Studienpopulation, die noch nicht vollumfänglich abgeschlossene Hörrehabilitation wie auch die breit formulierten Fragestellungen, welche die allgemeine physische und psychische Gesundheit betreffen. Andere Studien, welche ebenfalls allgemeine, subjektive Gesundheitsparameter untersuchten, zeigen ebenfalls ein unklares Bild. Bei gewissen Studien konnte eine Verbesserung festgestellt werden [14, 23], während andere unveränderte Werte zeigten [14, 22].

Im Gegensatz dazu verbesserte sich das subjektive Hörergebnis und Sprachverständnis bei einer Vielzahl von Patienten (16/25) nach Implantation wesentlich, insbesondere das Sprachverstehen in Lärm. Diese Befunde entsprechen den Befunden von publizierten Daten [5, 8, 28]. Die Verbesserung der Klang-Identifikation und Klang-Natürlichkeit braucht eine längere Adaptationszeit als 6 Monate. Die subjektive Höranstrengung ist ebenfalls in der initialen Phase der Hörrehabilitation nicht reduziert, da das Neu-Erlernen des Hörens mehr Ressourcen braucht. Längerfristig (> 1 Jahr nach Implantation) erwarten wir hier eine Reduktion [30]. Unser Ziel ist es durch die kontinuierliche Datenerhebung fortlaufende Normwerte für CI-Subpopulationen zu generieren. In Tab. 1 ist ersichtlich, dass bei unserer Studienpopulation Betroffene unter einer bilateralen, asymmetrischen oder unilateralen Hörstörung litten. Mit der Generierung von mehr Datenpunkten können diese Subpopulation einzeln analysiert werden. Unsere Daten, wenn auch von der Anzahl der Datenpunkte limitiert, zeigen vorerst keinen statistischen Zusammenhang zwischen der subjektiven Wahrnehmung des Sprachverstehens (wie sie im SSQ-12 erfasst wird) und der postoperativen Sprachaudiometrie im Freifeld. Das Erfragen der subjektiven Einschätzung bezüglich des Verstehens von Sprache kann deshalb wichtige Zusatzinformationen liefern.

Wir gehen davon aus, dass PROMs im Rahmen der Indikationsstellung insbesondere bei Individuen mit einer asymmetrischen oder unilateralen Hörminderung von großer Bedeutung sind. Dadurch können der Leidensdruck abgebildet und die Beratung entsprechend angepasst werden. In einer Studie konnte gezeigt werden, dass CI-Kandidaten mit einem niedrigeren SSQ-12-Wert eher bereit waren, einer Operation zuzustimmen und von einer Hörrehabilitation zu profitieren [27].

6 Monate nach Implantation nahmen die Ohrgeräusche bei über der Hälfte der Patienten (14/25) und im Mittel um 4,8 Punkte ab. Bei einer Minderheit nahmen die Beschwerden in diesem Zeitraum zu (um durchschnittlich 2,3 Punkte). Unsere Befunde stehen im Einklang mit publizierten Daten [20, 31].

### Einschränkungen

Die Studie konzentrierte sich ausschließlich auf erwachsene Teilnehmer. Die Einbeziehung von Kindern hätte aufgrund ihres Alters eine angepasste Auswahl der Instrumente erfordert und wäre durch die Abhängigkeit vom Ausfüllen durch die Eltern in der Aussagekraft eingeschränkt. Obgleich eine breitere Palette an Instrumenten und die Einbeziehung zusätzlicher Bereiche wie Gleichgewichtsfunktion für Erwachsene möglich gewesen wäre, lag der Fokus auf einer straffen Auswahl, um die Dauer der Befragung zu minimieren und sowohl die Antwortqualität als auch die Rücklaufquote der Fragebögen zu verbessern.

Zudem deckte die hier berichtete Datenanalyse den Zeitpunkt 24 Monate nach der Implantation unzureichend ab. Diese Daten müssen im weiteren Verlauf untersucht werden. Schließlich unterteilten wir die Hörstörungen in bilaterale, asymmetrische und unilaterale. Sobald mehr Daten vorliegen, sollte eine getrennte Subgruppenanalyse durchgeführt werden.

## Schlussfolgerung

In unserer Studie zeigt die erfolgreiche Integration von PROMs in der Behandlung erwachsener CI-Patienten. PROMs sind ein vielversprechendes Instrument zur Unterstützung in verschiedenen Behandlungsphasen, sowohl als Entscheidungshilfe für potenzielle CI-Kandidaten als auch zur Überwachung nach der Implantation. Unser Protokoll erfasst longitudinale Daten von der initialen Hörbeeinträchtigung bis hin zu frühen und späteren Rehabilitationsphasen, um die subjektive Behandlungsqualität zu beurteilen.

## Fazit für die Praxis

- Cochleaimplantate (CI) verfolgen 2 Hauptziele: Sie sollen die Lautsprachwahrnehmung wieder ermöglichen und die Lebensqualität der Betroffenen steigern.
- Aus diesem Grund sollten neben Sprachverständlichkeitstests patientenbezogene Ergebnisindikatoren (PROMs) systematisch erhoben werden, um die subjektive Wahrnehmung und Lebensqualität abzubilden.
- Geeignete PROMs müssen klar, relevant und in der klinischen Routine praktikabel sein.
- Die Integration von PROMs in den klinischen Alltag sollte für Patienten ohne wesentlichen Zusatzaufwand erfolgen, idealerweise elektronisch während bestehender Termine.
- PROMs sind ein vielversprechendes Instrument zur Unterstützung in verschiedenen Behandlungsphasen: sowohl als Entscheidungshilfe für potenzielle CI-Kandidaten als auch, um den Therapieerfolg individuell und umfassend zu beurteilen.
